# The suppression effect of emotional contagion in the COVID-19 pandemic: A multi-layer hybrid modelling and simulation approach

**DOI:** 10.1371/journal.pone.0253579

**Published:** 2021-07-28

**Authors:** Xudong Guo, Junbo Tong, Peiyu Chen, Wenhui Fan

**Affiliations:** Department of Automation, Tsinghua University, Beijing, China; Unviersity of Burgundy, FRANCE

## Abstract

The entire world has suffered a lot since the outbreak of the novel coronavirus (COVID-19) in 2019, so simulation models of COVID-19 dynamics are urgently needed to understand and control the pandemic better. Meanwhile, emotional contagion, the spread of vigilance or panic, serves as a negative feedback to the epidemic, but few existing models take it into consideration. In this study, we proposed an innovative multi-layer hybrid modelling and simulation approach to simulate disease transmission and emotional contagion together. In each layer, we used a hybrid simulation method combining agent-based modelling (ABM) with system dynamics modelling (SDM), keeping spatial heterogeneity while reducing computation costs. We designed a new emotion dynamics model IWAN (indifferent, worried, afraid and numb) to simulate emotional contagion inside a community during an epidemic. Our model was well fit to the data of China, the UK and the US during the COVID-19 pandemic. If there weren’t emotional contagion, our experiments showed that the confirmed cases would increase rapidly, for instance, the total confirmed cases during simulation in Guangzhou, China would grow from 334 to 2096, which increased by 528%. We compared the calibrated emotional contagion parameters of different countries and found that the suppression effect of emotional contagion in China is relatively more visible than that in the US and the UK. Due to the experiment results, the proposed multi-layer network model with hybrid simulation is valid and can be applied to the quantitative analysis of the epidemic trends and the suppression effect of emotional contagion in different countries. Our model can be modified for further research to study other social factors and intervention policies in the COVID-19 pandemic or future epidemics.

## Introduction

Since December 2019, the outbreak of the novel coronavirus, which later the World Health Organization (WHO) named as severe acute respiratory syndrome-coronavirus-2 (SARS-CoV-2), has affected the world tremendously. As the disease rapidly spread from one country to another, the WHO declared the COVID-19 pandemic on 11 March 2020 [1]. Up to 22 January 2021, SARS-CoV-2 has infected more than 97 million people [2]. Hence, it is quite essential to establish a model to simulate the transmission of the virus and provide useful guidance for public health decision-makers.

There are already many existing ways to model the process of disease transmission. For instance, the Susceptible-Exposed-Infected-Recovered (SEIR) model [3], is based on system dynamics modelling (SDM). With the simple structure, SEIR focuses on collective behaviors, which means all the individuals’ behaviors are supposed to be the same [4]. Lai et al. utilized the SEIR model to simulate and compare various non-pharmaceutical intervention methods adopted by China to contain the COVID-19 pandemic [5]. Besides, Chinazzi et al. [6] and Tian et al. [7] improved the traditional SEIR model and explored the suppression effect of traffic control on the COVID-19 pandemic. Though SDM has been widely used in epidemic modelling and analysis, individual heterogeneity is not considered because everyone is treated equally in SDM. What’s more, only the overall infected number can be obtained by SDM, thus the phenomenon of clustering outbreaks and the spatial distribution of the epidemic cannot be observed.

Another simulation method agent-based modelling (ABM) could overcome the problems mentioned above. In most of the existing literature, agent-based models take every individual as an agent, so that each agent can have its own attributes. The links between the agents can reflect the epidemic trajectory and the visualization of the agents makes it possible to illustrate clustering outbreaks. Perez et al. combined ABM with GIS technology to visualize the spread of infectious diseases in an urban environment [8]. And Cuevas et al. used ABM to evaluate the COVID-19 transmission risks in facilities [9]. However, the experiments in [8, 9] only considered a small population of fewer than 1,000 people, when it comes to large-scale problems, ABM may cost too much computation power, which is usually not affordable.

Whichever method is used to model the dynamics of epidemic spreading, most of the current models only concentrate on disease transmission, omitting the important effects of the human responses to epidemics. Emotional contagion is the spread of affect or behavior from one in a crowd to another, serving as negative feedback to the epidemic [10]. Some researchers have focused on the mathematical modelling of the mechanism of the emotion spreading itself [11–14]. Mahmud et al. have adopted SDM to model the emotional contagion in the COVID-19 pandemic but the disadvantages of SDM still exist [15]. A hybrid method combining SDM with ABM could be applied to a comprehensive model to introduce emotional contagion into the process of disease transmission. However, such a model is yet to be established and emotional contagion has not been considered in the analysis of the COVID-19 pandemic, which motivated the present study.

In this study, we proposed a multi-layer network model with hybrid simulation to simulate disease transmission and emotional contagion at the same time, thus these two parts made up of a closed-loop system, interacting with each other. By coupling SDM and ABM, hybrid simulation in our model provided the advantages of each approach: spatial heterogeneity and clustering outbreak phenomenon for ABM and larger simulation size and analytical solution for SDM [16, 17]. Our model can be applied to mega-cities with more than ten million people and finish the computation within a few minutes just using the ordinary CPU. Based on our model, we further analysed the suppression effect of emotional contagion in the COVID-19 pandemic among different countries. The model was implemented in Anylogic software.

The paper is designed as follows. Section II describes the proposed multi-layer network model and explains the hybrid simulation methods in detail. Section III presents the validation results to evaluate the performance of our proposed model and studies the effect of emotional contagion. Section IV illustrates our conclusions, the advantages and disadvantages of our proposed model as well as the future work based on our present study.

## Methods

### Data sources

We collected daily confirmed cases data of four cities in China: Guangzhou [18], Changsha [19], Nanjing [20] and Zhengzhou [21], from the official regional municipal health commission websites. Daily confirmed cases data of cities in the UK and the US were from the official UK Government website for data and insights on Coronavirus (COVID-19) [22] and COVID-19 Map by County and State from USA facts [23], respectively. We collected the population data of the cities in China, the UK and the US from National Bureau of Statistics of the People’s Republic of China [24], Office for National Statistics of the UK [25] and the US Census Bureau [26], respectively. The data have been cleaned and attached in S1 File.

### Multi-layer network model

**Fig 1:**
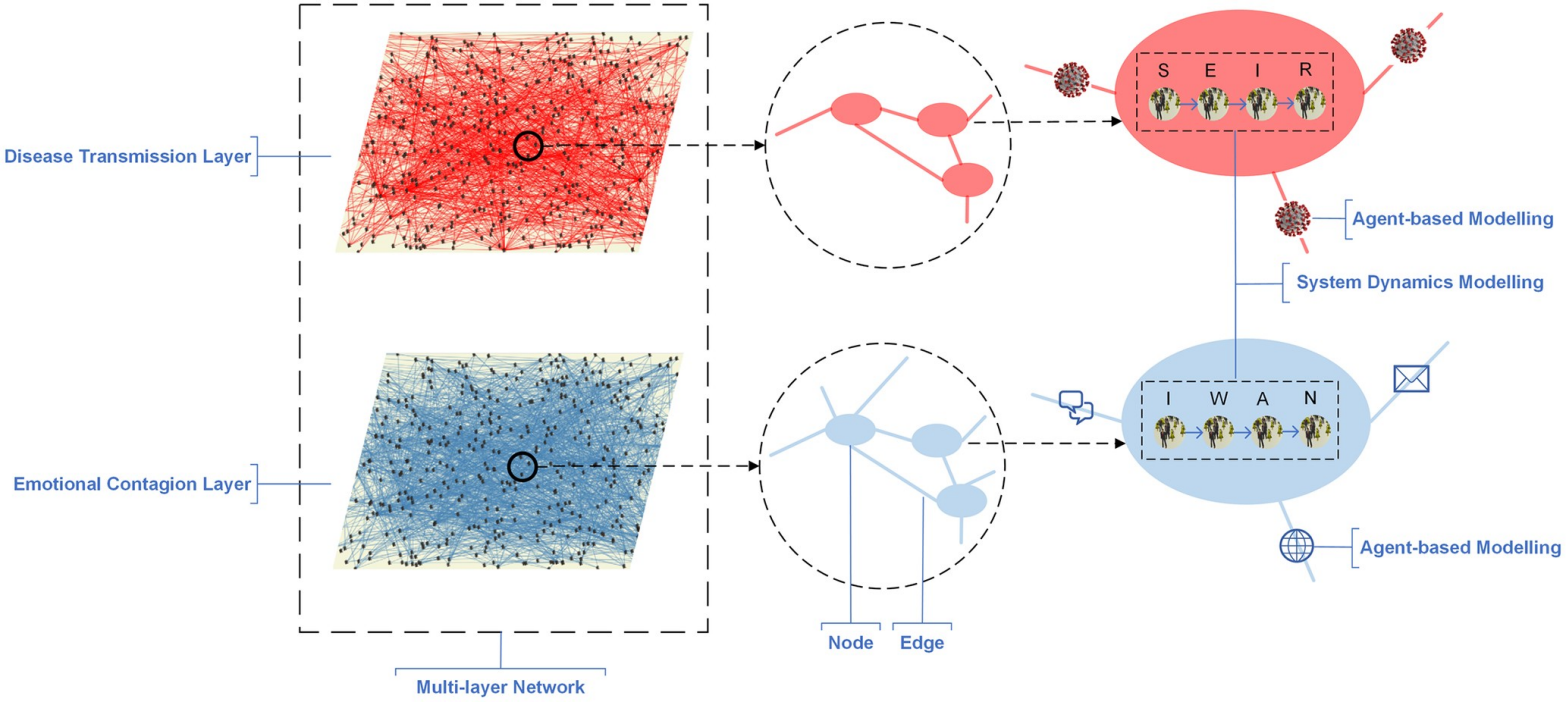
Overview of the multi-layer network model with hybrid simulation. SEIR (susceptible, exposed, infectious and recovered) and IWAN (indifferent, worried, afraid and numb) denote the states of the people inside a node. The nodes in the emotional contagion layer have more large-distance links than those in the disease transmission layer.

To independently analyse the spread of disease and emotion, we implemented a multi-layer network model to separate the two processes. Specifically, the two layers are the disease transmission layer and the emotional contagion layer. Each layer is a network and each node represents a group of people, such as a community, a university or a company, etc. Infection or information sharing happens through the edges between these nodes. The networks in the two layers are independent because the panic usually spreads by virtual links like the Internet instead of physical contacts. The whole structure of our model is shown in Fig 1.

### Hybrid simulation

Usually, a wave of epidemic starts with cluster infection. Likewise, rumor and gossip are usually spread in a small circle first [27]. Thus, it is necessary to apply different methods to model the global and local transmission. We used agent-based modelling (ABM) to simulate the disease transmission and emotional contagion between the nodes at the community scale. And within the node, system dynamics modelling (SDM) was adopted to calculate at the individual level so that the simulation would be more accurate and would not bring too much extra computational cost. People inside a node are separated into several subgroups due to their infection state or emotion state. The state will change as the epidemic gets severe, following the SDM models. Once the virus or the panic message is sent from one node to another, the receiving node starts the local transmission and becomes able to spread, too. The global and the local processes run in parallel.

### Agent-based modelling

**Fig 2:**
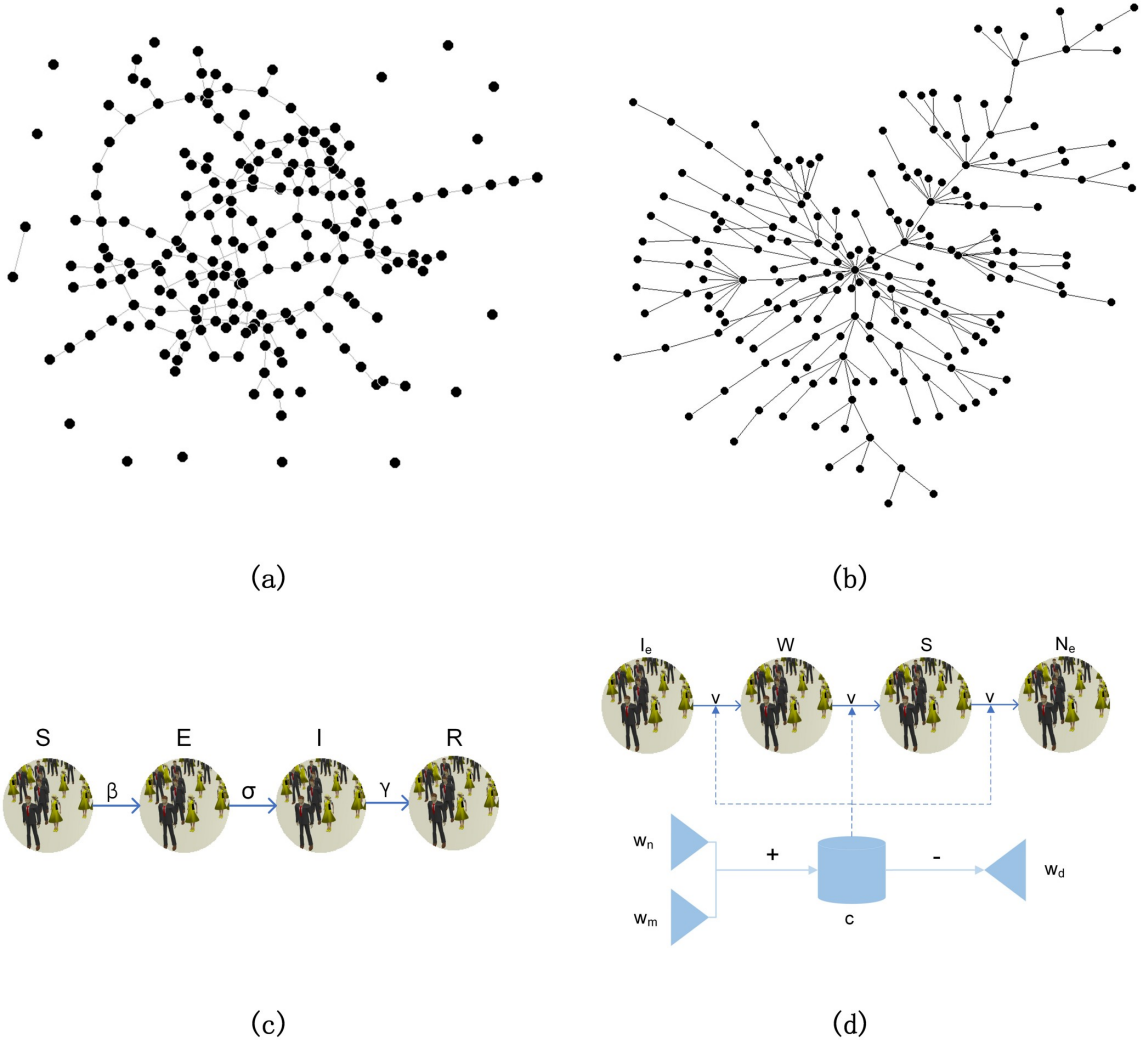
Illustration of the concepts and structures in our model. (a) Illustration of random network structure; (b) illustration of scale-free network structure; (c) illustration of SEIR (susceptible, exposed, infectious and recovered) model; (d) illustration of IWAN (indifferent, worried, afraid and numb) model. ‘+’ denotes increase and ‘-’ denotes decrease. Network visualization in (a) and (b) was done using the Pajek program for large network analysis [30].

Agent-based modelling was applied at the global level. Agents are connected bidirectionally to make up a network, thus each node in the network is an agent. If the nodes are linked randomly, as shown in Fig 2a, most of the nodes have a similar number of connections and the network is relatively homogeneous, which is not in accordance with our real world. According to Albert et al. [28], the scale-free network has higher heterogeneity, with few nodes connected by a lot of other nodes. The shape of scale-free networks is shown in Fig 2b. Approved by Albert et al. [28] and Grieshober et al. [29], connections in human society are usually fit to scale-free networks, like Facebook community networks. So in our model, the two networks in both of the layers are scale-free networks. Though the two networks are of the same type, they are independently generated and with different parameters, as there are distinct large-distance social interactions in the emotional contagion layer.

The interaction between the nodes in the two layers are similar. In the disease transmission layer, the node which has exposed or infected people inside is able to spread the virus to its neighbor nodes. We assumed that the neighbor was randomly chosen and as the time step of the simulation was one day, we assumed that the frequency of spreading was once a day. Similarly, the node with worried people can send panic messages to its neighbors to spread the emotion.

### System dynamics modelling: Disease transmission

System dynamics modelling was used inside every node. Given in Fig 2c, we applied the traditional SEIR (susceptible, exposed, infectious and recovered) model to the disease transmission layer. People in one node are separated into these four groups (S, E, I, R), and the state changes by Eqs (1)–(5):

$$\begin{array}{c} \frac{dS(t)}{dt}=-\frac{\beta S(t)I(t)}{N}, \end{array}$$ (1)

$$\begin{array}{c} \frac{dE(t)}{dt}=\frac{\beta S(t)I(t)}{N}-\sigma E\left(t\right), \end{array}$$ (2)

$$\begin{array}{c} \frac{dI(t)}{dt}=\sigma E\left(t\right)-\gamma I\left(t\right), \end{array}$$ (3)

$$\begin{array}{c} \frac{dR(t)}{dt}=\gamma I\left(t\right), \end{array}$$ (4)

$$\begin{array}{c} N=S(t)+E(t)+I(t)+R(t), \end{array}$$ (5)

Here, the exposed population (E) are the ones infected but asymptomatic, while the infectious population (I) refers to the symptomatic population. The dead people are included in the R state.

At first, all the population in a node are all in the S state. If the node receives the virus from another, then the SEIR transmission starts in this node: the exposed amount becomes one, and then more and more susceptible people in the node turn exposed, while the exposed turn infectious and then recovered. We list the notations used in the model in Table 1.

**Table 1:** Description of the notations used in our model.

Notation	Description
S, E, I, R	The states of disease transmission: susceptible, exposed, infectious, recovered
*β*, *σ*, *γ*	The infection rate, the incubation rate, the probability of recovery or death
N	The population of a node
I_*e*_, W, A, N_*e*_	The states of emotional contagion: indifferent, worried, afraid, numb
*v*	The transfer rate between the states of emotional contagion
*c*	The concern level in a node
*n*, *m*	The number of new confirmed cases in the city, the panic messages received by a node
*w*_*n*_, *w*_*m*_	The weights of *n* and *m* to calculate *c*
*w*_*d*_	The weight that *c* decays in a day
*w*_*c*_, *t*	The weight and threshold of *c* to calculate *v*
*r*_1_, *r*_2_	The reduction rate of *β* when one is in the worried state and afraid state

### System dynamics modelling: Emotional contagion

The emotional contagion layer serves as negative feedback to the disease transmission layer. The more panic one feels, the more actions will be taken to prevent infection, such as washing hands more frequently, putting on a mask, etc. More specifically, these actions will reduce the infection rate *β* in the local disease transmission. So how can we quantitatively represent the emotion and the contagion process of emotion?

As shown in Fig 2d, we introduced a new SDM model, IWAN (indifferent, worried, afraid and numb) model, to divide individuals in the node into four classes based on the magnitude of emotion, and set up the bridges for different classes to transfer [13]. The four classes are indifferent (I_*e*_), worried (W), afraid (A) and numb (N_*e*_). As long as the node receives a panic message from another node, this process will come into operation with the change of one individual’s state to W. Our IWAN model is given by Eqs (6)–(10):

$$\begin{array}{c} \frac{dI_{e}\left(t\right)}{dt}=-\frac{vW\left(t\right)I_{e}\left(t\right)}{N}-\frac{vA\left(t\right)I_{e}\left(t\right)}{N}, \end{array}$$ (6)

$$\begin{array}{c} \frac{dW(t)}{dt}=\frac{vA\left(t\right)I_{e}\left(t\right)}{N}+\frac{vW\left(t\right)I_{e}\left(t\right)}{N}-\frac{vA(t)W(t)}{N}, \end{array}$$ (7)

$$\begin{array}{c} \frac{dA(t)}{dt}=\frac{vA(t)W(t)}{N}-\frac{vN_{e}\left(t\right)A\left(t\right)}{N}, \end{array}$$ (8)

$$\begin{array}{c} \frac{dN_{e}\left(t\right)}{dt}=\frac{vN_{e}\left(t\right)A\left(t\right)}{N}, \end{array}$$ (9)

$$\begin{array}{c} N=I_{e}\left(t\right)+W\left(t\right)+A\left(t\right)+N_{e}\left(t\right), \end{array}$$ (10)

Different from the SEIR model in the disease transmission layer, here the transfer rate *v* is not a constant, instead, it varies as the virus spreads. To calculate the transfer rate *v*, we defined a new variable, concern level (*c*), to quantitatively describe the overall panic of a node. The relation between *v* and *c* can be defined as Eq (11):

$$\begin{array}{c} v=\{\begin{array}{c} w_{c}\log\left(c\right),c\geq t\\ 0,c<t \end{array}, \end{array}$$ (11)

Namely, if the concern level is lower than the threshold *t*, *v* will remain zero until the concern level is high enough. Then we assume *v* and *c* follow a logarithmic function, with a linear weight *w*_*c*_.

The concern level mainly comes from two sources: publicly available information (global) and information which comes from the social neighborhood (local). In our model, we chose two representative kinds of information as the sources: number of newly confirmed cases in this city (*n*) at the global level and panic messages spread from neighbor nodes (*m*) at the local level [31]. The corresponding weights for the two sources are denoted by *w*_*n*_ and *w*_*m*_. When the concern level increases because of the outside sources, it will also decrease as time passes by. We assumed that the concern level decreased uniformly by *w*_*d*_ every day. In sum, the concern level can be obtained by Eq (12):

$$\begin{array}{c} c=\int (mw_{n}+mw_{m}-w_{d})dt, \end{array}$$ (12)

Then we are able to design the feedback mechanism from emotional contagion to disease transmission. When there’s no interference, let the infection rate be *β*_0_. We assumed that people will put on masks in the worried state, reducing the infection rate by a constant *r*_1_. If people get into the afraid state, they are not going out unless necessary, causing a reduction of the infection rate by a constant *r*_2_. But people may get numb and relaxed as time passes by, and do not take actions to protect themselves anymore. According to Wang et al. [32], we set *r*_1_ as 0.2, and we assumed *r*_2_ as 0.02. In this way, we can get the updated infection rate *β*(*t*) by Eq (13):

$$\begin{array}{c} \beta \left(t\right)=\frac{\beta_{0}}{N}(I_{e}\left(t\right)+r_{1}\;W\left(t\right)+r_{2}\;A\left(t\right)+N_{e}\left(t\right)), \end{array}$$ (13)

## Results

### Parameter assumption

We list the assumptions in our model when conducting experiments on the actual data of the COVID-19 pandemic as follows. The infection rate *β*_0_ is 0.05249 according to Yang et al. [33]. The incubation period of SARS-CoV-2 is believed to be between 2 days and 14 days [34–36], so we assumed the incubation period $\frac{1}{\sigma }$ complies with a triangle distribution ranging from 2 to 14 with a vertex at 7. Similarly, we assumed the duration of infection $\frac{1}{\gamma }$ complies with a triangle distribution ranging from 7 to 28 with a vertex at 14. The initial susceptible population was set as the resident population and we assumed that there were 500 communities in a city, so the number of nodes in a network was set as 500. We assumed that the first exposed case appeared 7 days before the first infected case confirmed and we set the number of initial exposed individuals as three. We assumed that people who recovered would not get infected again.

### Simulation results in China

**Fig 3:**
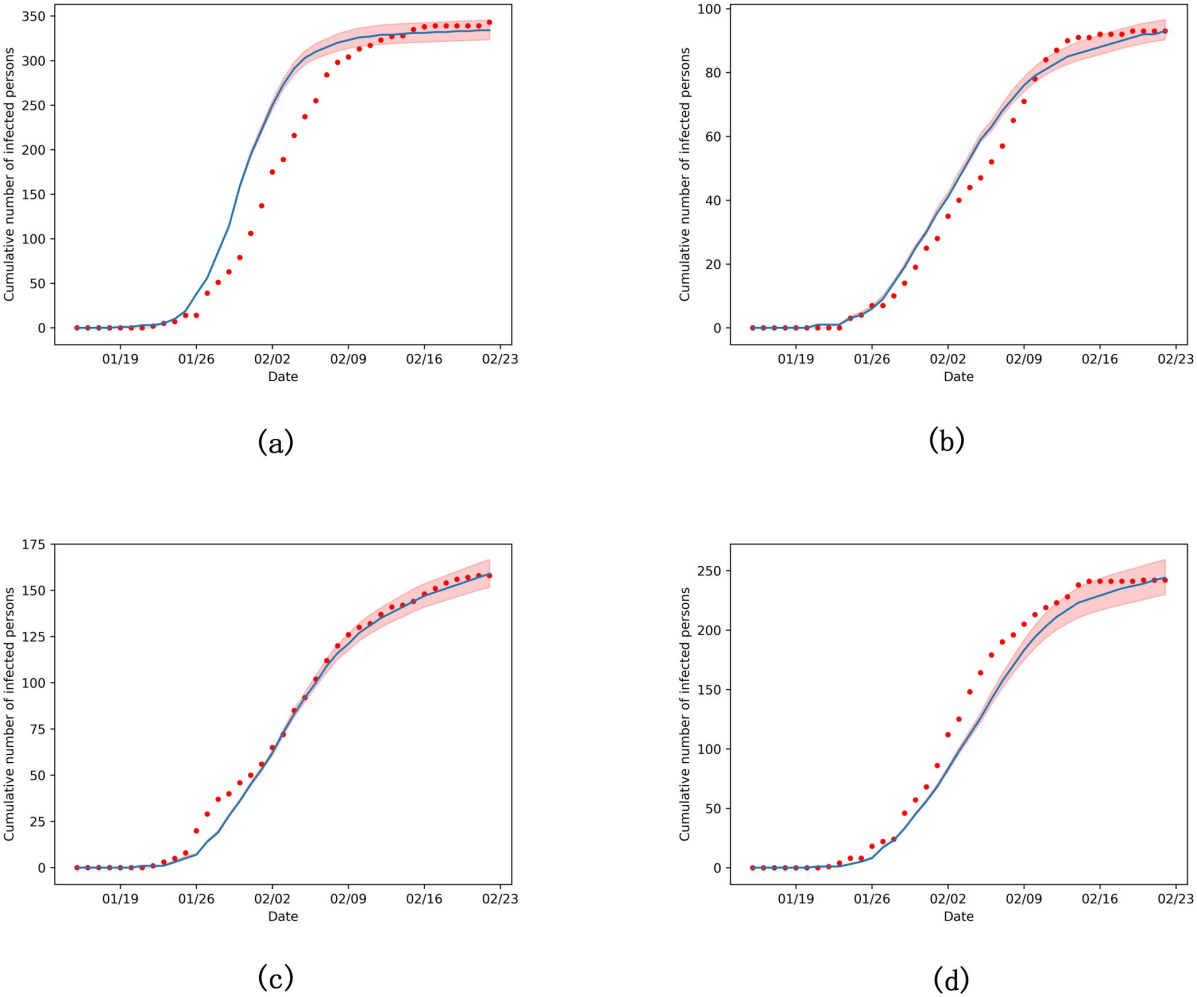
The cumulative number of infected persons in the cities of China. (a) Guangzhou, (b) Nanjing, (c) Zhengzhou, (d) Changsha. Actual data are fitted onto the curve (red circles).

Our model performed well on the actual data from China, here we illustrate the simulation results of several cities in Fig 3, including Guangzhou, Nanjing, Zhengzhou and Changsha. We ran the Monte Carlo simulations 100 times from 14 January 2020 to 21 February 2020, after which the spread of the virus was basically controlled. We also tested our model at the provincial level in China and the results are shown in S2 File. The patterns of simulation results are close to that of the actual data. In Table 2, we compared the peak of the cumulative number of infected persons during the simulation period with the actual data and we listed the 95% credible interval of the simulation results. It can be seen that our model basically reflects the epidemic trends and is valid on different scales.

**Table 2:** The peak of cumulative number of infected persons in the four cities of China: Guangzhou, Nanjing, Zhengzhou and Changsha.

City	Actual data	Simulation result
Guangzhou	345	334 (95% CI: 324–346)
Nanjing	93	93 (95% CI: 90–97)
Zhengzhou	158	159 (95% CI: 152–167)
Changsha	242	244 (95% CI: 230–259)

### Emotional contagion in different countries

**Fig 4:**
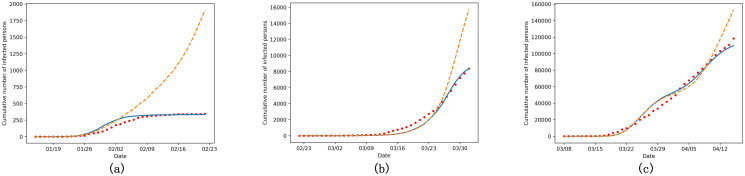
The suppression effect of emotional contagion. (a) Guangzhou, China, (b) London, the UK and (c) New York, the US. The solid lines denote simulation results with the emotional contagion layer and the dotted lines denote results without the emotional contagion layer. Red circles denote the actual data.

We also tested our model on the data from the UK and the US. The simulation duration was the same as the cities in China: 22 February 2020 to 1 April 2020 for London and 8 March 2020 to 15 April 2020 for New York. From Fig 4, we can see that the generalization of our model is good enough to suit different countries, even though the total confirmed cases and how people react to the epidemic may be quite different.

As analysed above, the emotional contagion layer slows down the original disease transmission. To verify the suppression effect, we conducted ablation experiments on the three cities in different countries, removing the emotional contagion layer while keeping all the rest parameters the same, shown as the dotted lines in Fig 4. Obviously, without considering the emotion, the simulation results would deviate greatly from the actual situation. The confirmed cases would grow exponentially and it took more time to reach the peak. For instance, the total confirmed cases in Guangzhou on 21 February 2020 grew from 334 to 2096, which increased by 528%. We can see that the suppression effect of emotional contagion cannot be ignored and it varies from country to country. This also leads us to think in another way, that it may be possible to quantitatively analyse the emotion variation by the parameters of the emotional contagion layer.

We compared the calibrated parameters in Guangzhou, London and New York in Table 3, and we got more evidence to prove that Guangzhou’s emotional inhibition is relatively more effective among the three, while New York’s is the least and London’s is between them. Take a look at the two sources of the concern level in the city, the weights of panic caused by new confirmed cases news (10.0) and panic messages from other nodes (10.7) in New York are less than those in London (19.7 and 29.7, respectively) and Guangzhou (13.9 and 128.9, respectively), which means people in New York comparatively are not scared of the pandemic. When considering the emotion spreading inside a node, the threshold for indifferent people to get worried (*t*) in New York is the highest (1049.0) while the transfer weight (*w*_*c*_, 0.2) is lower than the other two. So it may take more time for people in New York to feel worried and put on masks to suppress the virus.

**Table 3:** The emotional contagion parameters in Guangzhou, London and New York. The up arrow denotes that the higher the parameter is, the more effective the emotional contagion will be and vice versa. Here, *w*_*n*_ and *w*_*m*_ denote the weights of the number of new confirmed cases in the city and the panic messages received. And *w*_*c*_ and *t* denote the weight and the threshold of the concern level.

City	*w*_*n*_↑	*w*_*m*_↑	*w*_*c*_↑	*t*↓
Guangzhou	13.9	128.9	40.0	119.2
London	19.7	29.7	18.2	222.0
New York	10.0	10.7	0.2	1049.0

### Sensitivity analysis

**Fig 5:**
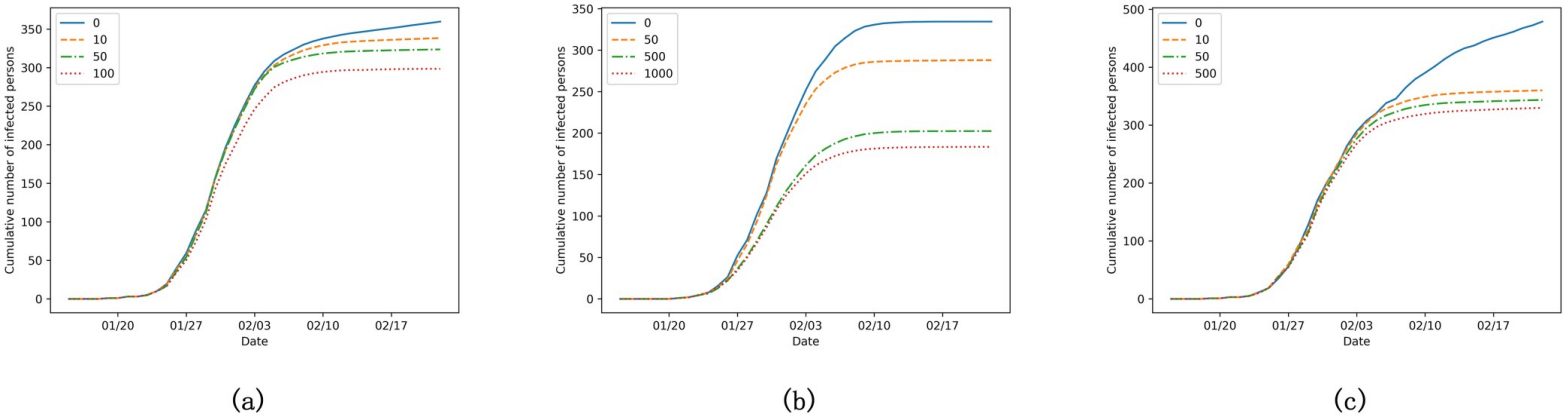
The sensitivity analysis of (a) *w*_*n*_, (b) *w*_*m*_ and (c) *w*_*c*_ in Guangzhou.

We also conducted a series of sensitivity analysis to understand the impact of changing emotional parameters on the simulation results. Take Guangzhou as an example, sensitivity analysis results of *w*_*n*_, *w*_*m*_ and *w*_*c*_ are shown in Fig 5. When keeping other parameters fixed, the increase of any of these three parameters would reduce the peak number of cases and flatten the curve. That the confirmed cases are sensitive to the emotional parameters also provides good evidence that the suppression effect of emotional contagion is notable and should be considered when modelling the epidemic.

## Discussion and conclusion

The outbreak of COVID-19 has caused great suffering to the human being. Due to the necessity and emergence of exploring the epidemic pattern, we proposed a multi-layer hybrid modelling and simulation approach to simulate disease transmission and emotional contagion together. Emotional contagion influences the self-protection degree of individuals and thus could suppress disease transmission. And the hybrid simulation method combines ABM and SDM to save computational cost while keeping the spatial heterogeneity, so our model can be applied to large-scale data, while the pure ABM is almost impossible for the high computation overhead. Besides, we designed a new emotion dynamics model IWAN (indifferent, worried, afraid and numb) to simulate emotional contagion inside a community during an epidemic.

With calibration, the simulation results of our model were close to the actual data. Based on the simulation and sensitivity analysis, we proved the effects of emotional contagion in the prevention of disease spreading. We also found that people in China are most influenced by the emotion to take self-protection measures, compared with the US and the UK. The parameters in our model could explain the difference among the countries from different aspects, including new confirmed cases news and panic messages from neighbors.

The findings in our study suggest that decision-makers should take advantage of the suppression effect of emotional contagion to control the epidemic. For instance, rapid epidemic notification systems should be established and people should be encouraged to spread objective and accurate epidemic news, so that emotional contagion may be more effective to suppress the disease.

Our model still has some shortages. We only chose two kinds of information as the sources of the concern level, and in fact, the computation of emotion is much more complex and there may be some other factors. Besides, we did not adopt the real community distribution data so there may be differences with the actual situation.

A future direction for this study could be conducting experiments of different interventions based on our model, such as restricting the traffic among the communities or quarantining the community with a confirmed case, etc. More factors influencing emotion could also be added into our model, e.g. the effects of media during the pandemic may be revealed using the multi-layer network model.

In sum, the proposed multi-layer network model with hybrid simulation is valid and could be applied to the quantitative analysis of the epidemic trends and the suppression effect of emotional contagion.

## Supporting information

S1 File Daily confirmed cases data of Guangzhou, Changsha, Nanjing, Zhengzhou, New York and London. (ZIP)

S2 File Simulation results at the provincial level in China. (DOCX)
