# Supplementary material for: The suppression effect of emotional contagion in the COVID-19 pandemic: A multi-layer hybrid modelling and simulation approach
Source: PLoS One. 2021 Jul 28;16(7):e0253579. doi: 10.1371/journal.pone.0253579 (PMC8318274; doi:10.1371/journal.pone.0253579)
Supplement: S2 File — (DOCX) [file pone.0253579.s002.docx]

**Simulation results at the provincial level in China**

We collected provincial daily confirmed cases data in China from the official website of National Health Commission of the People's Republic of China^1^ and the population data of the provinces in China from National Bureau of statistics of the People's Republic of China^2^. And we conducted experiments at the provincial level from 11 January 2020 to 21 February 2020. The results in Anhui, Guangdong, Zhejiang, Henan, Hunan, Jiangsu, Jiangxi and Shandong are presented in S1 Fig. It can be seen that our model can flexibly suit data of different sizes.


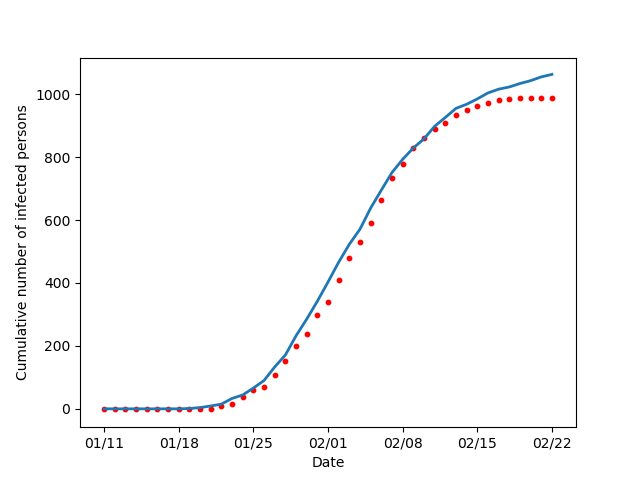

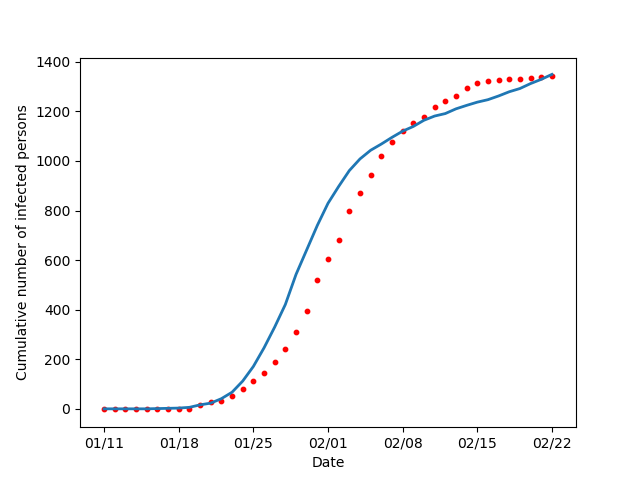


(a) (b)


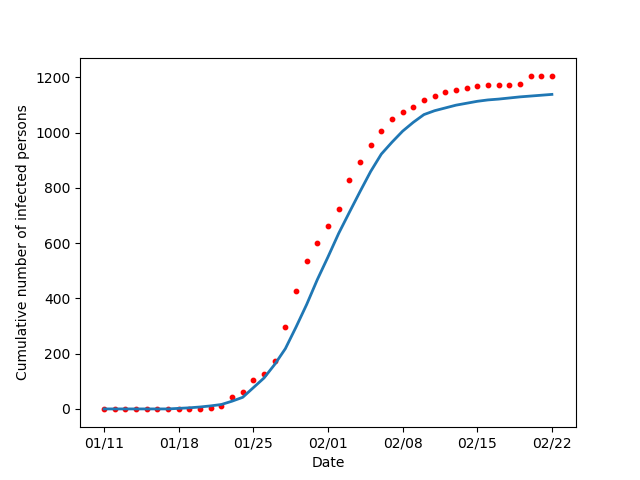

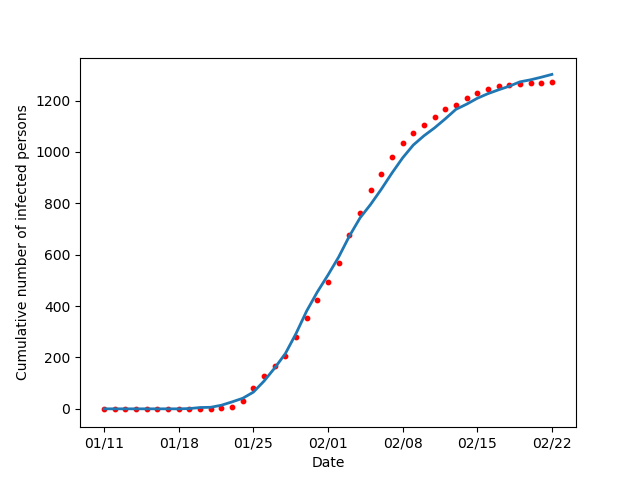


(c) (d)


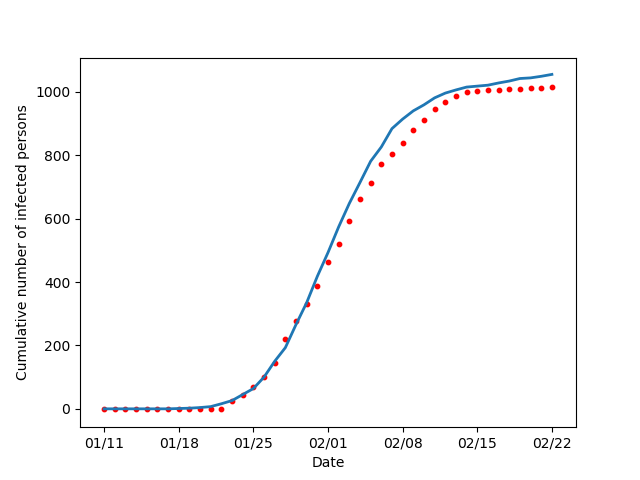

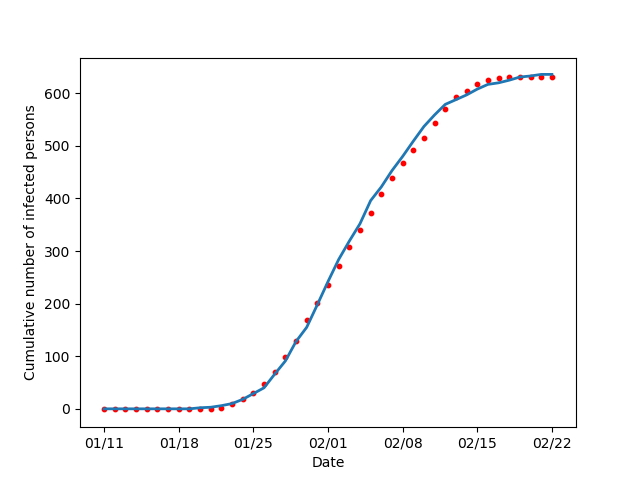


(e) (f)


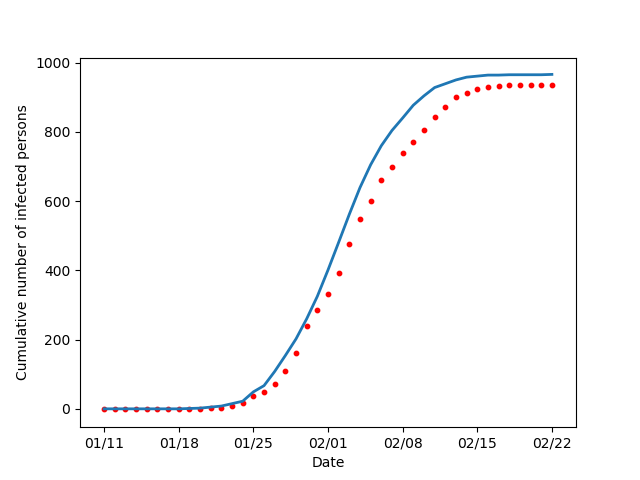

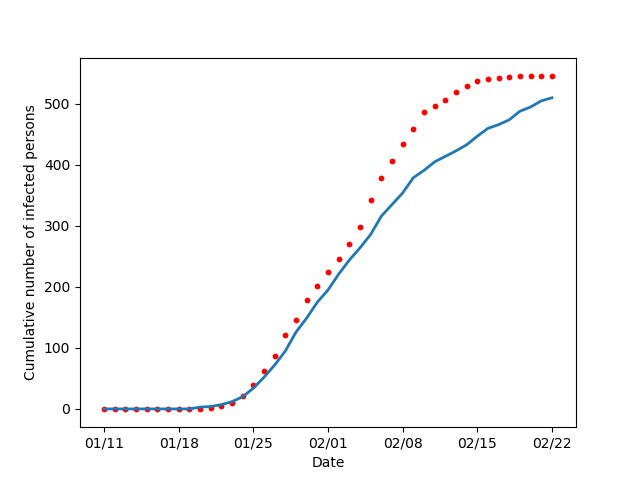


(g) (h)

**S1 Fig.** Cumulative number of infected persons in the provinces of China: (a) Anhui, (b) Guangdong, (c) Zhejiang, (d) Henan, (e) Hunan, (f) Jiangsu, (g) Jiangxi, (h) Shandong. Actual data are fitted onto the curve (red circles).

**Reference**

1. National Health Commission of the People's Republic of China. 2020. <http://www.nhc.gov.cn/xcs/yqtb/list_gzbd.shtml> (26 January 2021, date last accessed).
2. National Bureau of statistics of the People's Republic of China. 2021. http://www.stats.gov.cn (26 January 2021, date last accessed).
